# Algorithmic governance at work: a legal-sociological and policy analysis of AI regulation, institutional power and labor rights

**DOI:** 10.3389/fsoc.2026.1819161

**Published:** 2026-08-19

**Authors:** Anca Parmena Olimid, Cătălina Maria Georgescu, Daniel Alin Olimid, Silviu Dorin Georgescu, Cosmin Lucian Gherghe

**Affiliations:** 1 Political Sciences Specialization, Faculty of Social Sciences, University of Craiova, Craiova, Romania; 2 Biology Specialization, University of Craiova, Craiova, Romania; 3 Independent Researcher, Craiova, Romania

**Keywords:** AI, algorithmic governance, institutional power, labor rights, policy analysis

## Abstract

**Objectives:**

Artificial Intelligence (AI) has transformed and revolutionized the technological and informational space of the 21st century. AI has also reconfigured the way of relating and interacting with the information system, the work environment, the occupational structure, and the societal space. Within the framework of this study, the general objective aims to analyze and evaluate employee rights in the context of organizational change, technological innovation-driven algorithmic governance generated by automation and intelligent systems. The specific objective of the research focuses on reskilling under AI rules, AI civil liability for workers’ rights regime, ethical use of algorithmic governance at the workplace, and social and behavioral outcomes.

**Methods and methodology:**

The current research uses legal-organizational analysis (LOA), combining systematic literature review with legal and exploratory-descriptive study of the AI phenomenon in order to identify the current state of regulation in the field. This method is complemented by comparative legal analysis of different regulatory systems at a global level, as well as good practices in the workplace and case studies of representative examples of AI regulation in the workplace to identify how automation is transforming the occupational space and employment relationships.

**Results and discussions:**

The legal-organizational analysis highlights the legal developments in Europe, the Americas, and the Asia-Pacific, identifying the diverse dimensions of the regulatory architectural structure for AI technology across six interrelated pillars. The results indicate that AI regulation across Europe, the Americas, and the Asia-Pacific is increasingly intersecting around human rights and personal data protection, risk-based governance, and accountability mechanisms, particularly in the employment environment. Legal frameworks emphasize transparency, non-discrimination, and the need for human oversight in AI-powered workplace decisions.

**Conclusion:**

The conclusions highlight that across Europe, the Americas and the Asia-Pacific, AI regulation is increasingly organized around six dimensions: protection of human rights and data privacy in the workplace; governance frameworks addressing reskilling, job transformation and adaptation to AI-driven work models; risk-based oversight of AI systems; AI civil liability regimes affecting employee rights and HR practices; promotion of ethical AI to foster trust and mitigate bias; and strengthened monitoring, accountability and non-discrimination mechanisms.

## 1 Introduction

The study of artificial intelligence human-centric benchmarks casts and deepens the knowledge and research of regulatory policies, organizational advances, and strategic planning in the workplace. Integrating AI in the workplace stimulates and motivates the active workforce and drives a new positioning of the act of leadership and strategic work. The AI integration at the workplace positively impacts business, organizational, and operational efficiency (Keding, 2021). By integrating the digital and automated tools employed by AI, the employee’s usual processes are perfected, made more efficient, and improved work capacities at the workplace. However, this perspective engages another complex of variables in performing the routine tasks of employees, such as the dynamics of some factors of legal regulation of AI and other variables of the legal regime of technological innovation-driven algorithmic governance at the workplace, with an impact on employee performance, productivity, and individual satisfaction (Kassa and Worku, 2025).

The algorithmic governance use at the workplace implies the scrutiny, rationalization and logic of AI ethics with the anticipation and theorization of subsequent risks and security of AI and machine learning technology use (Nozari et al., 2024). The discussion about responsibility and control in algorithmic governance use at the workplace advances the examination of the rules and regulations of AI and their ensuing footprint on organizations’ management and personnel performance, data privacy and accountability. It also highlights the need to address how the broadening of digital technologies and algorithmic governance use at the workplace changes the organizational culture and operational effectiveness in various domains (Naji et al., 2026).

Hence, a discussion on employees’ engagement in the context of AI and digitalization augments and elevates a series of queries on managerial skills buildout, decision-making, and leadership (Liu and Song, 2022). Consequently, the investigation of the algorithmic governance use at the workplace raises questions about the design of enabling policies, strategies, institutions, and necessary surveillance for a human-centric perspective of AI use at the workplace theories and practice. The most recent literature provides an in-depth scanning of the regulatory process of technological innovation-driven algorithmic governance (Zaidan and Ibrahim, 2024). Thus, this study addresses the need for a constructive guide to organizations’ compliance with human-centric AI governance and the implications for entrepreneurial approaches. It builds on the conversations on engaging in the challenges and risks of AI and innovative technologies used at the workplace in the frame of reference of AI monitoring and oversight.

## 2 Literature review

How artificial intelligence (AI) and human-centric benchmarks approach are employed and regulated in the workplace have been in constant transformation in recent years, configuring an extensive conceptual framework transposed to several areas such as human rights, data privacy, data governance, ethical norms, risk assessment and implementation of AI systems, regulation of civil liability and employee rights, and use of AI and behavioral implications.

Although most specialized studies center on the human-in-the-loop research approach, focusing rather on the role of AI and favoring the discussion on productivity and smart manufacturing (Bhattacharya et al., 2023), production systems or intelligent production and efficiency (Abbas et al., 2024) to the detriment of human engagement and decision-making, a recent research direction labels other areas namely implementing an explainable AI (XAI) in human resource decision-making management (Dwivedi et al., 2023) by adapting the human-AI teaming approach at workplace (Pflanzer et al., 2023).

On the other hand, several studies manage both the dynamics in the human-centered approach, but also the feedback in AI management, involving discussions on inclusive leadership and sustainable development (Anane-Simon and Atiku, 2023). A new set of approaches favors a focused discussion on the nexus between organizational and legal changes, integrative leadership, and transparent management. In this context, Cheng et al. (2022) engage an integrative vision and a focused debate on the impact of AI on employees, highlighting the most important dimension of analysis for future research in the field, namely the influence of AI and employee reactions (Kaur and Sharma, 2021), as well as employee reactive mechanisms such as negative emotions (Hornung and Smolnik, 2022) and psychophysiological expressions (Lucifora et al., 2021).

Therefore, a key approach to the AI-M dimension (AI-driven management) is based on a double reasoning involving both organizational leadership and employees. In this context, Woodcock (2022) has approached AI implementation and its influence on organizational management and employee reactions (Link and Stowasser, 2024) by emphasizing new research directions on emotional response patterns (Zhang et al., 2024), new vectors and practices of high-performance work and organizational behavior (Kusmin et al., 2024), as well as a new focus engaging both psychological safety and organizational culture at the workplace.

Within the human-centric benchmarks, legislative changes must be sufficiently well-founded, clearly and objectively implemented, and have a clean and well-defined environment from an organizational, structural, and functional perspective. Such an approach therefore dimensions the role of inclusive management (Rambe, 2024), but also the workplace culture (Topic, 2025) by identifying those legal settings at the global and regional levels meant to redefine the human-centric landscape based on regulatory policies.

Thus, the present study aims to initiate and develop a framework for legal analysis of recently adopted legislative changes with immediate impact on the approach of algorithmic governance use at the workplace, centered on the role of the person in the workplace (Park et al., 2021). This approach emphasizes both the role of the principles of equity and responsibility, on the one hand, and the principles of decision-making transparency and organizational accountability, on the other hand (Syifa, 2024).

Following the latest legal regulations and organizational analyses, this study also highlights a human-centric approach and evidence from the perspective of the employee while undertaking a systematic cross-regional analysis. The study, therefore, focuses on the legal developments in Europe, the Americas and the Asia-Pacific, given the specific regulatory framework for algorithmic governance use at the workplace impacting the domains of (i) human rights, personal data and data privacy; (ii) artificial intelligence governance rules and impact on employees; (iii) AI systems and human risk-based assessment; (iv) AI civil liability for the employee rights regime; (v) ethical AI at work and behavioral outcomes and (vi) monitoring and oversight of AI use at the workplace.

## 3 Research design and methodology

In this study, we will use the legal-organizational analysis (LOA) method to employ a dual methodological approach (Roșca et al., 2020). First, we will examine the legal norms and regulations in force, and second, we will relate the legal framework to the practices of algorithmic governance use at the workplace. The objective of applying LOA is threefold: first, LOA is justified by the need to correlate the legal norms in force with AI-assisted management. Second, LOA has the role of assessing and monitoring legal compliance, but also to monitor the observance of rights and ethical principles. Third, LOA integrates and assesses the legal dimension with organizational management and managerial accountability in the context of digitalization.

As a qualitative research method, LOA uses comparative legal analysis that allows the exploration of organizational issues in the workplace due to the impact of AI in different contexts. Subsequently, LOA develops a supplementary approach based on the possibility of direct observation of the legislative impact of AI implementation by collecting and analyzing legislative changes. The analysis of the updated legislative framework allows the establishment of relationships between legislative approaches regarding the use of AI in the workplace, as well as the management of risks in the sphere of employee rights. The legal aspects of AI implementation in organizational culture engage and associate with the assessment of legal compliance of norms under the most recent legislative updates regarding the protection of personal data.

Therefore, for the selection of legislation to be analyzed, we consider three complementary and cumulative readings.

(1) The first criterion concerns the geographical location and typology of the legal system, reflecting the following directions of analysis:
(i) Common law: United Kingdom, United States of America, Singapore, Canada, and Australia. For the specific American legislation, both federal initiatives and sub-federal acts regarding the regulation of AI in the hiring and promotion process were selected as follows: Public Law No. 113-128 (United States Congress, 2014); Executive Order No. 13859 (United States Government, 2019); Public Law No. 116-283 (United States Congress, 2020); Executive Order No. 13960 (United States Government, 2020); Local Law No. 144 (New York City Council, 2023); Australia’s Artificial Intelligence Ethics Principles (Australian Government, 2024).
For Singapore, two initiatives were selected for analysis based on skills development (Government of Singapore, 2015) and the jobs-skills integrators (Government of Singapore, 2023). These two initiatives, launched by the Singapore government, address the role of vocational training in the context of digital transformation and AI with the aim of identifying industry partners and improving placements and the development of training programs.
In the case of Canada, three documents adopted between 2022 and 2025 were analyzed, which cumulatively address the role of legal regulation, professional development, and adaptation to labor market requirements. The three initiatives highlight the role of AI regulation (Government of Canada, 2022). while maintaining the focus on adapting the skills ecosystem, education and training, but also on increasing productivity, Government of Canada (2025a), in the context of technological and digital changes, Government of Canada (2025b).
(ii) Civil law: France and Switzerland. The selection of the French model is justified by the existence of a system of collective rights of employees that confers and guarantees the freedom to choose their professional future. In this context, Law No. 2018-771 regulates newly registered rights, including the right to training independent of the employment relationship. This regulation becomes a solid protection mechanism in the context of AI (République française, 2018).
(iii) Legal reflections at the European Union level. Regulation (EU) 2024/1689 represents the global reference point that establishes the binding legal framework underpinning risk classification.
(2) The second criterion aims to select legislation according to the degree of maturity and crystallization of the normative provisions. In this context, preference was given to legal systems in which the regulation of the implementation of AI in the context of employment relationships was achieved at the level of adopted and in force normative acts, either in the form of a normative framework that is being built progressively, but which has progressive legal force, but with mandatory vocatives. The two documents selected for analysis fall into this last category. The first is entitled “OECD principles on artificial intelligence” (2019), a document of a guiding nature, but whose normative provisions have a significant legal and political importance regarding the development of public policies at the international level. The second document is Regulation (EU) 2024/1689, which is in the phase of progressive implementation at the level of the Member States of the European Union, recognized by comparative doctrine and jurisprudence as a normative pillar of legislative convergence.

The research approach, therefore, positions six research foundations (section 4. Results and discussions), namely the reporting on employee rights and compliance with labor law principles amid AI-driven organizational change (subsection 4.1.), reskilling under AI rules (4.2.), AI systems and human risk-based assessment (subsection 4.3), AI civil liability for employee rights regime and the need to identify regulatory gaps (4.4.), ethical AI at the workplace and behavioral outcomes (subsection 4.5.), ethical AI at the workplace as well as a critical reflection of legal considerations in the matter of personal data confidentiality (4.6.).

The six research directions were developed in Section 4. Results and discussions represent elements of the convergence of three sources of analysis. The first inductive source derives from the systemic reading of the normative acts selected according to the criteria specified above, which led to the delimitation of the six analytical categories (from 4.1. to 4.6.). The second doctrinal and axiological source is based on the selective legal and sociological literature highlighted in the second section of this research, intended to operationalize additional analysis plans, such as operationalizing the analytical categories, legitimizing the social relevance of the topic addressed, and providing the empirical framework of the applicable legal norms.

## 4 Results and discussions

### 4.1 Upholding employee rights amid AI-driven organizational change

The adoption in April 2024 of the European Union Artificial Intelligence Act (EU AI Act) marks an important stage in the relationship between the regulation of the protection of employees’ rights and the implementation of algorithmic governance use at the workplace, given the provisions of Annex III (European Parliament and Council of the European Union, 2024). According to Annex III of the EU AI Act, employees’ rights require precise and express regulation of high-risk AI systems used for critical infrastructure, education, and vocational training in the stages of learning, training, and professional assessment, and employment concerning the process of recruitment and selection of the workforce. The EU AI Act also addresses the organizational transformation and access to essential public services through AI systems implementation by safeguarding employee rights amid AI systems used in migration management, asylum monitoring, and border control management, and AI systems used in trials and administration of justice.

The new EU regulation sets a framework for respecting fundamental human rights in the workplace, including the protection of rights and data, the principle of non-discrimination, decisional transparency, autonomy, and human dignity. In terms of regulating employers’ obligations, the new EU regulation sets a four-fold mechanism for managing and assuming obligations for high-risk AI systems by implementing a risk management system (Article 9 of the EU AI Act), maintaining efficient management for documentation and registration, post-implementation monitoring, and clear and well-defined organizational management of resources.

In comparison, in the United Kingdom, although there is no single legal framework regulating the use of AI in the workplace, there are two regulations governing indirect discrimination resulting from the use of algorithms, particularly in recruitment and performance assessment processes (Equality Act, 2010) and processing data subjects’ rights (Data Protection Act, 2018). The British legislation adopted in 2018 with the Data Protection Act (DPA) provides a clear and individualized regulatory basis for lawful processing and respect for the principle of transparency in the processing of personal data, imposing the obligation on employers who use AI systems for monitoring to have a concise legal basis and a commitment to guarantee data confidentiality (Data Protection Act, 2018). The DPA 2018, therefore, provides a legal basis for the right to access and explanation in the process of accessing data collected by AI systems and provides for human intervention in the case of automated decisions with a direct impact on the employee (Sections 45, 49, and 50, DPA 2018).

In the United States of America, the lack of a particular single framework governing the regulation of AI in the workplace is compensated for by the adoption at the federal level of two new executive orders defining the national strategy in the field of AI.

The first order refers to the impact of AI on the labor market and calls for the need for upskilling and encouraging educational and training initiatives and professional adaptation for employees in the context of the implementation of Executive Order No. 13859/2019 (United States Government, 2019). The second order commits a triple commitment to the responsibility of government employees who must use AI systems that respect workers’ rights addressing an institutional-managerial responsibility on the part of agents that obliges to avoid abuses and illegal use of AI systems in the process of supervising the implementation of AI, as well as a multisectoral assessment of professional rights and risks as stipulated by the Executive Order No. 13960/2020 (United States Government, 2020).

If at the federal level, the regulatory provisions of recent years have mainly employed organizational leadership accountability and management-level duties and obligations, other normative acts adopted at the local level have configured new applied and integrative legal mechanisms, dimensioning other central frameworks for the respect of employee rights, namely: notification of potential employees regarding the use of an automated tool in the process of evaluating the qualification for the job, the limited territorial and jurisdictional scope of regulation and application, the purpose of implementation regarding the hiring and promotion process and the legal regime of sanctions and institutional responsibility in case of violation of the provisions.

Such a normative act, recently adopted by the city of New York, Local Law no. 144, entered into force on July 5, 2023, and explicitly regulates the “automated employment decision tools” (AEDT) sector in the administrative code of New York. The legislative proposal previously introduced in February 2020 was approved on November 10, 2021, and entered into force in July 2023 after the completion of regulatory procedures. The AEDT represents a software system based on both the implementation of artificial intelligence and on algorithms, used in the workplace in the process of recruitment, selection, and hierarchization of candidates or employees. To protect employee rights, the newly introduced regulation for the AEDT sector is based on guaranteeing transparency and accountability in employment relations with application to the procedures of recruitment and selection of candidates, but also to the specific processes of promotion of employees (New York City Council, 2023).

The most important provisions newly introduced by the AEDT legally regulate the system of software based on artificial intelligence and applied in decision-making management at the workplace in New York City as stipulated by § 20-870, Subchapter 25 of Local Law no. 144 (New York City Council, 2023). The newly adopted legal framework configures an institutional governance supported by a bias audit and a system of notification of applicants and employees within at least ten business days regarding the use of an automated tool in the evaluation process (Article § 20-871, Local Law 144/2023). The information must highlight the qualifications and characteristics of the evaluation tool. A significant aspect of the new AEDT regulation focuses on the right of applicants and employees to opt for other alternatives in the recruitment or evaluation process (Articles § 20-872, § 20-873, and § 20-874, Local Law 144/2023).

In Canada, with the launch in June 2022 of the new AI regulation entitled “Artificial Intelligence and Data Act” (AIDA) (Government of Canada, 2022), which directly regulates the provisions applied to the professional and organizational framework of activities, although the AIDA provisions do not explicitly use the term “workplace.” However, the new Canadian legislation mentions a new regulatory framework with a direct focus on high-impact systems used in professional activity at the workplace in sectors and stages of professional life explicitly mentioned by the legislator, such as the recruitment and selection system, but also other professional stages, such as the promotion or dismissal of employees. AIDA sets a risk assessment system in the management of AI implementation that refers to the decision-making process and employee rights based on the principles of non-discrimination and equity. AIDA exemplifies the typology of systems with potential impact, such as screening systems used for services or activities at the workplace, as well as biometric identification systems used by certain AI systems (Government of Canada, 2022).

A major contribution to the regulation of AI in the workplace was adopted in Switzerland in November 2020 (Swiss Federal Council, 2020), stating from the first level of proposals (Guideline 1) the protection of human rights and dignity, as well as the need for constant monitoring of developments in the field of AI.

At the same time, Swiss legislation regulates the legal framework of rights and obligations in the workplace secondarily through the regulation in the Swiss Code of Obligations (CO) by expressly regulating the contractual obligations for both parties, employer and employee. Although the CO does not regulate explicit provisions regarding the impact of AI implementation in the workplace, certain regulatory provisions are nevertheless intended to affect the contractual obligations by the legal principle of good faith, the right to contest, as well as contractual and tort liability (Articles 41 and 97, Swiss Confederation, 2023a).

Other indirect regulations regarding the data protection regime of employees in the event of the implementation of an AI system, the need to inform employees regarding the use of an AI system in the event of employee evaluation, but also the obligation of explicit consent of employees in expressly specified situations are regulations established by the new provisions recently adopted by the regulations provided for by the new Federal Act on Data Protection (Swiss Confederation, 2023b) which entered into force on September 1, 2023.

According to the latest Report of the Swiss Federal Council entitled “Overview of artificial intelligence regulation” launched on 12 February 2025, the new strategic framework guarantees human rights and protects personal data by the principles and legal norms regarding transparency, data protection, and non-discrimination (Part 1, Swiss Federal Council, 2025). The Swiss overview report (2025) engages a risk-based approach to federal AI guidelines, assuming the public and private domains equally.

### 4.2 Reskilling under AI governance rules

After analyzing the legal framework regarding the protection of employee rights at work, it is also important to analyze retraining policies, especially in areas and countries that emphasize new policies and effective strategies for retraining and professional adaptation.

With the technological and digital advancements generated by artificial intelligence, workplace governance rules have configured new automated work tasks and have engaged in an innovative trend adapted to new skills. In the new framework of requirements generated by the automation of mechanisms and processes in the workplace, both employers and employees have adapted recurrently by engaging in retraining, as a process of adaptation and learning to respond to the need for change (Markose et al., 2026). Retraining and reskilling have become a constant in the transition process to a new field of activity, revealing three complementary mechanisms, namely the transformation of jobs, the automation of work tasks, and the design of AI governance rules in managerial practices, both in the field of personnel recruitment and with regard to the remodeling of the method of employee selection.

In this context, the need to update the legal framework by approaching the retraining and the reskilling policies becomes equally important as the technological transition advances and new opportunities are launched for employees affected by automation.

For the European Union, the Pact for Skills (European Commission, 2020) and the European Social Fund Plus (European Commission, 2021) specify the retraining of jobs generated in the context of artificial intelligence by employing a solid legal framework of policies and regulatory principles based on continuous training and retraining, partnerships for the development of digital skills and the development of new opportunities for upskilling and professional reconversion. Both documents promote the principle of fair governance in the workplace in the context of changes induced by the technological transition, with an emphasis on access to training and the acquisition of new digital skills and new abilities.

Beyond sector-specific regulations for reskilling and upskilling, financing policies also play an important role in the transition from one field to another. A comparative normative analysis reveals that the activation and implementation of reskilling financing policies vary considerably depending on the field of employment, but also on the social and economic context.

Therefore, in France, beyond the differences in regulation at the institutional level, there are several convergent aspects regarding employees’ rights to training, as well as the possibility of establishing and setting up a Personal Training Account (Compte Personnel de Formation – CPF) in accordance with the provisions of labor law (République française, 2018). French legislation regulates professional mobility and lifelong learning and guarantees the individual’s right to training, being applicable and extended to unemployed people in order to access and finance reskilling programs during active life.

The novelty of the CPF mechanism established by French legislation is that it allows the financing of vocational training courses for both professionally active people and the unemployed, but also for other categories of workers and young people who have left the educational system without obtaining a diploma. The purpose of the program is to support new professional skills, to develop and improve mobility and professional retraining, as well as to obtain a professional certification within the limit of a financial ceiling established by the French legislature, which is also the holder of the right to regulate the CPF mechanism. The grants accessed through the CPF mechanism allow access to training programs to acquire a qualification, and obtain new skills and professional competencies, but also support for validating experience and assessing skills in the labor market.

Although the regulatory initiatives are similar in the United States, the federal law adopted in 2014 establishes labor market adaptation programs ensuring collaboration between federal institutions exercised at the level of government agencies and employers (United States Congress, 2014). The new regulatory framework aimed to coordinate the functional mechanisms and to design support for employees to acquire new skills in the context of AI implementation. Another complementary perspective for the analysis of AI governance rules concerns the “National AI Initiative Act of 2020,” which establishes a triple institutional mechanism to respond to the challenges brought by the technological transition by supporting policy priorities in the field, investments in research and education, as well as professional training program initiatives (United States Congress, 2020).

On the other hand, in Singapore, the governance framework regarding reskilling and upskilling policies fundamentally engages a new type of commitment based on programs such as SkillsFuture (Government of Singapore, 2015) which allow the granting of individual grants to young people up to the age of 25 to encourage access to professional development, lifelong learning framework and completion of programs to improve skills and develop competencies by encouraging public-private partnerships, but also the Jobs-Skills Integrators initiative within the budget for 2023 (Government of Singapore, 2023), a pilot project for reskilling, hiring support and workplace analysis.

At the beginning of 2025, in the context of the implementation of automated systems and processes generated by artificial intelligence, two programs and projects of the Government of Canada were launched within the Future Skills Center (FSC) initiative to evaluate and identify new policies and strategies in workplace governance in the context of the advancement of technological transition. The specificity of the first initiative refers to the impact analysis on the productivity of companies and firms (Government of Canada, 2025a). The second initiative is designed as a pilot project based on the integration of an inclusive AI in the skills development portfolio, but also in the professional skills progression ecosystem (Government of Canada, 2025b).

### 4.3 AI systems and human risk-based assessment

The rapid transformations of the World Wide Web, exponential increase in digital market services, and growth in AI systems and applications for personal or professional use, have accelerated the need for a fresh revision and governmental intervention on AI ethics and algorithmic governance use at the workplace rules along the dimensions featuring processed data, algorithmic machine learning (ML) technologies and AI systems (Schneider et al., 2022).

The appraisal of AI rules and regulations, especially in organizations, implies looking into privacy settings, management, and personnel duties and work returns. However, using AI technology and machine learning in different work-related fields implies liabilities, ensuring mechanisms within the work culture and functioning authority at certain levels. The spread of AI technologies in a diversity of work dimensions and living-related aspects has generated a concern over the risks involved. Research has focused on different risks associated with the early operation and use of AI systems and applications in work-related fields, urging political decision makers to counterbalance AI advantages with a closer scrutiny of AI risks (Büthe et al., 2022). In light of these risks, policymakers face the need to address and contain possible consequences, gaps, and challenges (Taeihagh, 2021). Next, the literature selection presents a variety of AI systems and technologies risks.

#### 4.3.1 Fairness, equity, and lack of error

A first category of algorithmic governance use at the workplace risks refers to unfairness, inequity, and algorithmic systematic error, where different aspects and areas of fairness in AI judgment and decision-making in workplaces have been linked to human perceptions of “organizational justice” (Narayanan et al., 2023). Concerns over lack of fairness, often pointing towards “algorithmic bias,” influence perceptions of organizational justice within the human-technology nexus relating to the companies’ system of appreciation and decision-making process (Kordzadeh and Ghasemaghaei, 2021). Maintaining a balanced and mindful approach at the workplace as regards the innovations to HR functions and the use of generative AI applications proves key (Ghosh et al., 2024).

In this respect, the EU AI Act addresses different dimensions of biased outcomes and discrimination consequences (Recital 27, Regulation (EU) 2024/1689), especially as regards the necessity to regulate the human „remote biometric identification” (Recital 32, Regulation (EU) 2024/1689).

#### 4.3.2 Clarity and intelligibility

Another risk of algorithmic governance use at the workplace raises concerns over clarity and intelligibility, especially when dealing with human resource development (HRD) and career development as ethical issues, data correctness, private life, and decision communication and understanding are key (Ghosh et al., 2024). Studies have shown the advantages for HR managers of using AI prediction and interpretation AI models of employees’ behavior and career choices; however, coupled with the transparent translation and contingent understanding of the data returned (Chowdhury et al., 2022).

#### 4.3.3 Transparency and explainability

Transparency concerns, as well as raising awareness on the visibility and informed consent of human-AI interactions, with their inherent limitations, and binding rules for high-risk AI systems are addressed in the EU AI Act (Recital 26 and Recital 27, Regulation (EU) 2024/1689). The regulatory frameworks have to observe research findings on the occurrence of different dimensions of ethics at the workplace and follow a human-centric approach on the safe and transparent use of AI in companies, businesses, and different areas of services (Tlili et al., 2024). Also, the literature has raised the question of the limits and limitations of explainability in AI or “eXplainable AI” (XAI) in high-risk areas such as healthcare and medical diagnostic technologies (Onitiu, 2023), police control and justice administration, finances and credit rating (Giudici and Wu, 2025) while EU initiatives have introduced these issues raising awareness over careful regulation, meticulous monitoring and ubiquitous application (Pavlidis, 2024; Dear, 2019).

#### 4.3.4 Observance and safety

Another concern addressed the issue of observance and safety in the use of AI from a human rights perspective, where privacy considerations and security mechanisms represent essential assessment items (Cocito et al., 2025).

#### 4.3.5 Liability, reporting, and control

Moreover, concerns over regulating liability, reporting, obedience, and control as regards algorithmic governance use at the workplace, and considering a framework of accountability and responsibility for the specific manifest roles of AI systems (Lindgren, 2024). Concerns over automated decision systems at the workplace within high-risk environments have, for instance, been addressed in the California Workplace Technology Accountability Act, which limits the time, exposure, context, and control of AI use for the benefit and safety of workers (Hilliard et al., 2022).

#### 4.3.6 Job replacement

Furthermore, employees’ and experts’ concerns over the risks of human replacement have been addressed through studies that urge the adoption and growth of skills in critical areas of expertise, but also approach development and improvement (Jaiswal et al., 2021).

Consequently, the discussion about emerging skills acquisition for both management and execution employees with different areas of prowess in the AI dawns is also connected to the adoption and implementation of qualifying policies, assessment, monitoring, and human oversight for a human-centered approach of AI use at the workplace.

The EU’s intentions to develop a dominant position in AI regulation in its quest for digital sovereignty have been manifest in its AI Act enactment within its quest for resilience and for a harmonized framework of AI rules and digital rights. Studies hence research the intentions and scope of such an endeavor, considering the interests of European citizens across the EU entrepreneurial environment (Mügge, 2024). Consequently, filling the gap on artificial intelligence regulation and addressing the emerging risks ensures effective and ethical AI governance across both private and public organizations (Butcher and Beridze, 2019; Henman, 2020).

Further, human outlook and appraisal of AI risks and corporate compliance with AI governance and legislation are important factors for stakeholders and policymakers in influencing employee behavior and boosting organizational competitiveness (Albalawee and Fahoum, 2024).

Also, studies over authoritative regulation databases suggest that the efficiency of AI governance relies on linking technological advances to mindful regulation that focuses on sound ethical principles and trustworthiness of AI (Sharma, 2023).

Within the EU, the AI Act defines “AI systems” and “machine-based” systems, “deployer,” “biometric data,” “biometric identification,” “biometric categorization,” “remote biometric identification system,” “emotion recognition system,” “publicly accessible space,” “informed decisions,” “high-risk activity,” “high-risk AI system,” “trustworthy and ethically sound AI.”

Furthermore, the EU AI Act is designed to efficiently regulate AI systems and human risk-based assessment against manipulation, discrimination, data, and bias that infringe EU fundamental values and democratic principles (Recital 28, Regulation (EU) 2024/1689).

These rights and principles are also enshrined in the European Commission’s *European Declaration on Digital Rights and Principles for the Digital Decade,* designed along a human-centered approach of human rights and fundamental values and freedoms as a reference mark for corporate and government officials (European Commission, 2022).

### 4.4 AI civil liability for employee rights regime

Artificial intelligence governance rules and impact on employee rights have grown into a profound interest for both academia and policymakers in studying and regulating AI systems regimes for their effects on organizations and employees. Separate dimensions of explainable AI systems (XAI) are studied as particular situations to assess the results and trust perceptions in human-automated systems within work-related circumstances defined as “situation awareness framework” (Sanneman and Shah, 2022). Further, the “situation awareness framework” provides a human user experience-centered approach styled to mitigate liabilities for employee advantage and soften decision-making processes (Jiang et al., 2022).

Hence, within the management of risk literature and sociological research, AI risks fall as liabilities to human rights, fundamental freedoms, democratic values, and trustworthiness commitments. Personal data calibers in the EU AI Act include, though are not limited to the design of AI regulatory sandboxes for security in different sectors, healthcare, environmental and energy considerations (Article 59) and further in high-risk AI system trials (Article 60), human oversight of AI is regulated (Article 14) though the question of trustworthiness of AI remains a serious concern (Kusche, 2024).

In search for solutions against AI use risks, the study of Human-Centered AI (HCAI) in contrast to Human-Centered Design approaches brings into focus the questions over motivation or intention, ethics or morals, and resources or securities in line with the conversation of the European Commission propelled “high-level expert group on artificial intelligence” (AI-HLEG) where the accent to the fortune of natural persons and society (European Commission, 2022; Schmager et al., 2025). Further, as in the case of companies, the use of AI in education environments (Akinwalere and Ivanov, 2022), for assessment or selection purposes, for instance, encounters numerous risks and perils which have to be addressed through a combination of methodologies and by imposing regulatory institutions at different authority levels (Luo and Zhang, 2025; FRA, 2018). The imposition of human-centric AI systems has been invoked in the literature which defined human centrism and argued for a clear and effective regulatory insertion, where ethics, fairness and human well-being thus stand at the foreground of these conceptual approaches targeting the following dimensions: incorporating human wellness, robust accountability, bound to confidentiality, embodies the human centrality postulates, responsibly regulated, monitored and controlled, following deliberate analytical appointments (Ozmen Garibay et al., 2023; FRA, 2020).

Arguing the need to introduce into regulation the definition, scope and mechanisms of AI human centric approach, studies highlighting the insertion of human oversight for high-risk AI systems into the EU AI Act in Article 14 for the time in which the systems are applied as a “safeguard” against risks, ethical breaches and malware by prescribing the object of oversight, the period and the accountable entity (Enqvist, 2023).

### 4.5 Ethical AI at the workplace and behavioral outcomes

Connecting the discussion over ethical AI in the workplace to the argument over the monitoring and oversight of algorithmic governance use at the workplace. Beyond the added value of resorting to AI in decision-making processes based on data analysis, data processing, and predictions, the concerns over ethics, data privacy, accountability, and algorithms’ risk and manipulation raise the need for governmental action to develop sound AI regulation to cover loopholes in legislation or unregulated corporate governance aspects (Kumar, 2024).

Studies have shown the influence of using artificial intelligence in contrast to emotional intelligence at the workplace on employees’ performance and probability to increase job retention (Prentice et al., 2019), the influence on employees’ innovation and turnover compared to the effects of leadership (Tahir et al., 2024), the transaction costs impact in human resource management (Pan et al., 2021). Studies have analyzed the combined effects of employing AI and strategic objective management practices and models in securing organizational performance (Mahboub and Ghanem, 2024; Akinwalere and Chang, 2024). AI effects over management, however, seem to differ, while routine tasks such as documentation are necessary in analytical, reflective activity are compensated through AI, specific human managerial leadership innovative skills remain highly present (Giraud et al., 2022). Studies on employee engagement supplement within the HR cooperative activities using AI-operated chatbots have shown their contribution to employee satisfaction (Dutta et al., 2022; Wang et al., 2024). Government practices and public administration traditions are prompted by regulatory proceedings that need adequate insurance mechanisms against public risks (Wirtz et al., 2020; Roberts et al., 2022).

National strategies drafted by governments on artificial intelligence (AI) advocate for control of AI from a hybrid governance angle, and also include both public and private perspectives, crafting new monitoring and oversight of AI institutions (Radu, 2021: pp. 178–193).

Studies have shown that executives, financial observers, and investors rely on data analysis, AI ethics principles regulated and implemented in companies thorough going AI to support their financial corporate decisions (Brusseau, 2021). Studies on the use of deep learning AI on employees and companies have suggested implications for work development and career choices that stem from gaining understanding and creativity in human resources development (Li and Yeo, 2024) realizing in some cases a coaction conjunct beneficial unity between AI and human resource development assignments (Khandelwal et al., 2024) which often translates into increased revenues and ability to solve certain nuisance, although there are numerous questions and concerns one has to wonder and balance (Budhwar et al., 2022; Sepasspour, 2023). The ethical disquietudes and risk apprehension of people analytics use in different human resources management functions cover an increase in studies on efficiency and performance-related logic of AI use at the corporate level (Giermindl et al., 2021). A similar responsibility-driven approach surrounds studies on correlative approaches between business analytics and AI employment powered by contingency dimensions, especially in terms of data regulation and governance, and resource uniqueness, including employees’ traineeships and proficiency (Rana et al., 2021; Djeffal et al., 2022). Consequently, researchers positioned within the responsible logic of AI use and AI “dark side” scrutiny, assessment, and study (Mikalef et al., 2022). The controversy in applying innovative human resource development (HRD) AI-intensive functions is alleviated with AI schooling competences’ presence (Li and Kim, 2024), while even HRD per se sometimes encourages and advocates for AI functions acquisition and appropriation (Khandelwal et al., 2024), especially when including a far-reaching set of AI ethical guidelines in the process (Wang and Pashmforoosh, 2024). Studies have proven that generative AI (GenAI) in itself has not contributed solely to employee work performance, but rather a combination of confidence and usability or friendliness has directly impacted work enthusiasm and ingenuity (Marimon et al., 2024). Likewise, artificial intelligence systems are more likely to be exploited in work contexts associated with a serviceable or pragmatic purpose (Jo and Park, 2023).

### 4.6 Creating ethical AI at the workplace

Given both levels of regulation and implementation of the legal norms and provisions, the practical approach to reskilling and upskilling must rely on ethical principles at the workplace. In this context, an important recommendation was launched in 2019 within the framework of the initiative entitled “OECD principles on artificial intelligence,” which recommends that national governments encourage and stimulate innovation through AI while also guaranteeing compliance with ethical and social principles in the safe and ethical sharing of data (Organisation for Economic Co-operation and Development, 2019).

Also, to complete the legal framework in the field, the European Commission adopted a new legislative guide in 2019 (European Commission, 2019) based on ethical principles for the implementation and use of AI in the workplace, by the norms and principles of technical security, personal data protection, decision-making transparency, fairness, and legality. The legislative act adopted in 2019 complements the norms and provisions in the field adopted under the General Data Protection (GDPR) (European Parliament and Council of the European Union, 2016), as well as the EU AI Act (2024).

The principles set out in the GDPR explicitly focus in Recital 104 on the reference to “protection of human rights” and “international human rights norms” and are based on transparency under Recital 59, Article 5, and Article 88 (European Parliament and Council of the European Union, 2016), accountability in the case of data protection impact assessments, fairness and non-discrimination in the process of managing personal data under Article 5 and Article 18 (European Parliament and Council of the European Union, 2016). Also, Article 22 GDPR guarantees the rights of employees in automated processing processes if these processes significantly affect their professional life (European Parliament and Council of the European Union, 2016).

Other recent regulations primarily substantiate the principle of transparency for informing employees by requiring companies to operationalize an audit in the case of using automated hiring systems (New York City Council, 2023) or for reporting situations of algorithmic discrimination if the system arbitrarily favors certain socio-professional categories. Other relevant requirements for compliance with the legal and ethical spectrum refer to the obligation to conduct an independent audit, respecting transparency by publishing the audit results on the employer’s website, and ensuring fairness in the management of AI systems.

In Australia, a set of government guidelines was launched on 7 November 2019 for the responsible management of AI implementation, and updated on 11 October 2024, focusing on voluntary principles in the context of AI implementation. These principles commit both to focusing on human norms and values such as the well-being of citizens, human values, the protection of privacy, as well as the management and operationalization of artificial intelligence systems with respect for the principles of transparency, accountability, safety, contestability if an AI system has a significant impact on a person, group or society as a whole (Australian Government, 2024).

In the United Kingdom, the set of guidelines introduced by the AI Regulation White Paper (Department for Science, Innovation and Technology, 2023) details and commits to a fair and transparent regulatory framework for implementing an ethical AI ecosystem in the workplace. The foundation of the new regulation is both the ethical use of AI and a framework of cross-sectoral principles designed to address the risks associated with the operationalization of AI in a transparent, explainable, and proportionate manner. As can be seen from Table 1, the legal and public policy sources highlighted in the research are systematized considering the thematic relevance related to the implementation and use of AI in employment relationships.

**Table 1:** Comparative table of specific regulations on the use of AI in the course of employees’ work, reflecting the parameters of the countries under comparison and the national legislation adopted.

Jurisdiction	Source/Legal instruments	Year of adoption	Thematic relevance for the use of AI in the course of employees’ work
Australia	Australian Government (2024). *Australia’s Artificial Intelligence Ethics Principles*. Australian Government.	2024	AI ethics and principles
Canada	Government of Canada (2022). *Artificial Intelligence and Data Act*, as part of *Bill C-27, Digital Charter Implementation Act, 2022*, 1st Sess, 44th Parl, 2022	2022	AI regulation; Employee rights: right to transparency regarding high-impact AI systems; right to protection from discriminatory automated decision-making
Canada	Government of Canada (2025a). *Waiting for Takeoff: The Short-Term Impact of AI Adoption on Firm Productivity* [Future Skills Center].	2025	Right to be informed on workforce planning; Right to equitable distribution of AI productivity and benefits
Canada	Government of Canada (2025b). *Unleashing AI into the Skills Development Ecosystem* [Future Skills Center].	2025	Right to reskilling; Skills development plan and policies; Right to equitable distribution of AI productivity and benefits
France	République française (2018). *Law No. 2018–771 of 5 September 2018 on the freedom to choose one’s professional future*	2018	Right to personal training account (CPF); Right to skills development
United Kingdom	Department for Science, Innovation and Technology (2023). *A pro-innovation approach to AI regulation: White Paper*.	2023	AI regulation in the workplace; Right to accountability for automated decisions
United Kingdom	Equality Act (2010), c. 15. (UK).	2010	Right to non-discrimination in AI-assisted hiring tools
Singapore	Government of Singapore (2015). *SkillsFuture: Empowering individuals to take ownership of their skills development*	2023	Right to reskilling for career progression
Singapore	Government of Singapore (2023). *Budget 2023.* Ministry of Finance Singapore	2023	Right to training during digital transformation in the workplace
Switzerland	Swiss Confederation (2023a). *Swiss Code of Obligations (CO)*. Classified compilation 220.	2023	Right to contractual protection and guarantees concerning: risks associated with AI system use Right to civil liability in the context of the use of AI systems
Switzerland	Swiss Confederation (2023b). *Federal Act on Data Protection (FADP)*. Classified compilation 235.1.	2023	Right to data protection; Right to transparency and right to access personal data.
Switzerland	Swiss Federal Council (2020). *Artificial intelligence in Switzerland – Ethical guidelines and position of the Swiss Federal Council*.	2020	Right to human dignity and right to address a human oversight when AI systems generate adverse consequences
Switzerland	Swiss Federal Council (2025). Report of the Swiss Federal Council.	2025	Right to regulatory protection and participation in AI governance and implementation policies in high-risk industries
United States of America	New York City Council (2023). *Local Law No. 144 (Int. No. 1894-A)*	2023	Right to notice the AI use in the recruitment, hiring, or promotion process
United States of America	United States Congress (2014). *Workforce Innovation and Opportunity Act* (Public Law No. 113–128). U.S. Government Printing Office.	2014	Right to a personal career development plan
United States of America	United States Congress (2020). *National AI Initiative Act of 2020* (Public Law No. 116–283). U.S. Government Printing Office.	2020	Right to preparation for AI transition
United States of America	United States Government (2019). Executive Order No. 13859, 3 C.F.R. 3967 (2019).	2019	Right to preparation for AI transition guaranteeing human development
United States of America	United States Government (2020). Executive Order No. 13960, 3 C.F.R. 78939 (2020).	2020	Right to accurate and safe AI while interacting with AI systems
European Commission	European Commission (2019). *Ethics guidelines for trustworthy AI*. High-Level Expert Group on Artificial Intelligence	2019	Right to fairness Right to non-discrimination
European Commission	European Commission (2020). *Pact for Skills: Opportunities for reskilling and upskilling*. *Pact for Skills*	2020	Right to reskilling and upskilling
European Commission	European Commission (2021). *ESF+ 2021–2027: Investments in education and training*. *ESF+*	2021	Right to education and training. Right to digital skills development.
European Commission	European Commission (2022). *European Declaration on Digital Rights and Principles for the Digital Decade* COM/2022/28 final	2022	Right to dignity in AI-mediated environments
European Commission	European Parliament and Council of the European Union (2016). *Regulation (EU) 2016/679 of the European Parliament and of the Council of 27 April 2016*	2016	Right of the data subject not to be subject to a decision taken solely by automated means
European Commission	Regulation (EU) 2024/1689 of the European Parliament and of the Council of 13 June 2024 laying down harmonized rules on artificial intelligence	2024	Right to prior notification of high-risk AI use

Source: Table elaborated by the authors based on the legislations cited within the present research.

Table 1 reflects accelerated legislative changes in the last decade aimed at responding to a triple challenge generated by the exponential evolution of algorithmic innovation, the responsiveness of the legal system of regulation and adaptation, but also by the need to form a global standard of response regarding the protection of employees’ rights, right to preparation for AI transition, right to reskilling and upskilling, right to human dignity.

At the level of analysis of regulatory similarities and functional convergences, Table 1 reflects eight distinct jurisdictional systems (Australia, Canada, France, the United Kingdom, Singapore, Switzerland, the United States of America, and the European Union) with a specific taxonomy of regulation and governance based on the existence of regulatory principles and objectives.

At the level of common principles, the eight jurisdictional systems stipulate and converge around responsibility, ethics, transparency, non-discrimination, explainability and fairness, as well as the protection of fundamental rights of the person.

At the same time, Table 1 projects a complete regulatory spectrum, and the normative convergences of the selected jurisdictions reflect a relative uniformity of the legislative reforms regarding the emerging standard regarding the protection of employees’ rights, notification in the case of the use of AI systems and the risk-based logic operationalized by the AI Act.

At the level of regulatory differences, the jurisdictional systems present normative variabilities that dedicate distinct normative solutions differentiated in terms of regulatory intensity and the obligations established. This framework includes in a first phase the normative finality: mandatory and horizontal in the case of the European Union and voluntary and sectoral in the case of Singapore and the United Kingdom.

Within the European Union, Table 1 reflects regulatory strengths focusing on three regulatory models, namely France, Switzerland, and the United Kingdom. The regulation in the Swiss jurisdiction privileges international law, such as the right to data protection, the right to transparency, and the right to access personal data.

In this context, Table 1 reflects a normative asymmetry of regulations generated mainly by legal traditions and the source of the norm (member state status with the application of European Union law, international treaty for Switzerland, or state mosaic in the case of the United States of America and Canada).

Although the eight regulatory systems aim at comparable purposes, they differ mainly in the normative techniques used and the legal effects produced. In essence, the British legislative option enshrines sectoral principles such as the right to human dignity, the right to accountability for automated decisions, and the right to non-discrimination in AI-assisted hiring tools.

In this framework, France offers a regulatory model that complies with the AI Act, but which supports the individual right to training, attached to the person, not the workplace.

The particularity of the regulation of the French system resides both at the institutional level through the construction of an expertise infrastructure for compliance with the AI Act, but also at the individual level, at the level of the rights registered in the personal training account that remain validly acquired in the event of a change in professional situation or in the event of job loss. The French model focuses on the socio-economic effects of work, anticipating through the CPF the mutations and transformations on the labor market in the context of technology. At a practical level, the French regulation strengthens the role and rights of the individual to training and retraining.

Therefore, for the evaluation of the effectiveness of regulations, there are three complementary dimensions, namely normative relevance, institutional framework of implementation, and social implications. We can conclude that the regulatory normative framework, but also the institutional regulatory framework legislated by the EU AI Act (2024), as well as the regulations in French legislation, concentrate the most solid package of rights and guarantees for employees, through expressly mentioned and regulated obligations, sanction mechanisms, and specialized institutions and instruments with expressly provided powers and competencies. At the same time, other legal systems, such as Singapore and Switzerland, have not developed institutional structures with equivalent powers.

In the third dimension allocated to social implications, two major perspectives remain unregulated, but they may be the subject of future national legal regulations in the following areas:

(i) The employee’s right to challenge automated decisions with an impact on workplace relationships. Although Article 22 GDPR provides for a general right of the data subject not to be subject to a decision taken solely by automated means (European Parliament and Council of the European Union, 2016), the effective application of this regulation is unclear given the risks of legal interpretation regarding the concept of “exclusively automated,” as well as a complex and extensive interpretation of automated processes including the different stages and actors in the decision-making chain.
(ii) The right to collective bargaining. Article 26(7) of the EU AI Act regulates the notification and information of employees regarding the use of AI systems at work. In addition, the legal provisions in force do not grant trade union organizations the right to intervene in the employer’s decision-making process. This aspect translates into a major gap and a legal cleavage between the obligation of transparency and collective consultative participation, with only the obligation of prior information and notification of representative organizations before implementation.

Critics have expressed concerns regarding the risks of excessive regulatory intervention on innovation in AI technologies (Ayub, 2024). As a consequence, AI systems have had the opportunity to be tested under regulatory sandboxes, envisaged as supervised testing environments that encourage innovation of AI technologies under regulatory support, raise awareness on legal requirements before market implementation, and are designed to offer thorough monitoring and wide surveillance for risk detection (Truby et al., 2022). Moreover, regulatory sandboxes are able to create a collaborative environment for legislators, AI tech companies, academia, and civil society representatives. For instance, at the EU level, under the EU AI Act, each Member State has to develop its own AI regulatory sandbox (Plato-Shinar and Godwin, 2026). However, there are also reservations in using regulatory sandboxes before the release of massive AI technologies, as critics argue that within this context, participating companies endowed with sufficient financial and technical resources, as opposed to small or medium-sized enterprises, may exert their influence over legislators. Further, participating businesses may present the impression that their technology is compliant, approved, and thus safe for users (Ahern, 2025), when in fact AI use in employment or other contexts could pose severe risks and effects to fundamental rights. Another concern stems from the fact that sandboxes allow a diversity in regulations and standards, which highlight risks of adopting uneven or even contradictory AI legislation in different countries (Novelli et al., 2025).

Consequently, new concerns arise regarding the challenges created by the need to accommodate transnational regulatory convergence efforts to domestic legal frameworks and context-specific legal autonomy. The fragmented application of AI sandboxes rules across national jurisdictions can determine changeable legislation over time that addresses the contextual specificity and legal autonomy across countries (Zarra, 2026). Another concern deals with the probability that sandbox participation monitoring and supervision absorb more financial and expertise resources than are available, or that it expands beyond the regulatory deadlines due to technical matters (Yordanova and Bertels, 2023). There are even concerns that public scrutiny cannot engage during the supervision process, thus limiting its public transparency or visibility (Buocz et al., 2023).

The discussion thus addressed the need to consider filling the regulatory loopholes in the national laws of the countries studied and the EU legal framework regarding the algorithmic governance use at the workplace and employment practices (Bokaei et al., 2024; Lund et al., 2025).

The analysis of contemporary regulatory trends reflects not necessarily a convergence of regulatory models but rather a prudent regulatory divergence focused on flexible solutions adapted to the institutional context, designed to respond clearly, urgently, and quickly to rapid developments and transformations in the field. The limits of the research remain rather structural and are mainly related to the accelerated pace of algorithmic innovation and the temporality of legal regulations in the context of the asynchrony between the guarantee of employees’ rights at work and market competitiveness.

In addition, the practical effectiveness of legal provisions is analyzed and reflects both different intensities and degrees of normative regulation, but especially various models of legislative implementation. From the horizontal model adopted with the AI Act, based on the increased protection and guarantee of rights, to the sectoral models exposed by the systems in the United Kingdom and Singapore, we find that the practical effectiveness of regulations becomes flexible and adapts depending on the institutional and economic context, but also on the framework for guaranteeing rights at work.

In this context, the role of the application of the legislative framework and the temporal dimension of regulations becomes an essential factor for the real efficiency of legal mechanisms, illustrating the need for an adapted infrastructure of legal governance based on clear legal norms, regulatory authorities, and a governance regime based on enforcement mechanisms and real implementation capacity.

The research results thus indicate that governments have envisaged rather context-adapted solutions, as there is no unique regulatory framework governing AI in the workplace. Consequently, the following policy recommendations are voiced to address technology innovation and economic needs with workers’ rights.

Policymakers and regulators should prioritize the design and enforcement of effective regulatory frameworks backed by institutional mechanisms of AI oversight and scrutiny, constantly refined and improved to balance technology innovation and organizational practices, empowering independent regulatory bodies and monitoring authorities, and offering policy direction for employers and companies.

Employers, companies, and other stakeholders should expand their focus on AI governance beyond legal compliance to cover algorithmic governance, organizational policies’ impact and risk evaluations, human oversight, employees’ involvement in the decision-making process, and regular training on AI technologies and digital support.

## 5 Conclusion

Building on an extensive literature review doubled by an intensive documentation of regulatory instances, this study performs a highly analytical legal-organizational analysis (LOA) on a human-centric algorithmic governance use at the workplace. The management of AI systems under the provisions of human risk-based assessment implicitly relies on evaluating the rationale of AI ethics while deducing the security risks in using AI technology and machine learning in different personal, work, private, public, or professional environments. The arguments for algorithmic governance used in workplace regulation and control indicate the question of accountability and responsibility while performing under organizational efficiency prerequisites. Ethical outcomes in terms of ethical human-AI interactions uphold the following dimensions: proficiency, probity, trust, duty and obligation, human rights and propriety deference, social security and comfort, and corporate sustainability.

The comparative research underlines the legal developments in Europe, the Americas, and the Asia-Pacific, highlighting the intrinsic dimensions of the regulatory architectural structure for AI technology and systems of the following dimensions.

(i) Human rights, personal data, and data privacy, discussing the regulation of employees’ rights amidst the employment of AI at the workplace.
(ii) Artificial intelligence governance rules and impact on employees, arguing for the reskilling and retraining of employees, job remodeling and transformation, adapting and learning in the context of a new work framework.
(iii) AI systems and human risk-based assessment discussing governmental intervention on AI ethics, privacy settings, monitoring, and minimizing associated risks.
(iv) AI civil liability for the employee rights regime, discussing the impact on human resource management and employees’ turnover.
(v) Ethical AI at work and behavioral outcomes, arguing for mitigating risks.
(vi) Monitoring and oversight of AI use at the workplace, deepening the discussion on the principles set out within national and international legislation on the protection of human rights, accountability, and non-discrimination.

The analysis of contemporary regulatory trends reflects not necessarily a convergence of regulatory models but rather a prudent regulatory divergence focused on flexible solutions adapted to the institutional context, designed to respond clearly, urgently, and quickly to rapid developments and transformations in the field. The limits of the research remain rather structural and are mainly related to the accelerated pace of algorithmic innovation and the temporality of legal regulations in the context of the asynchrony between the guarantee of employees’ rights at work and market competitiveness.

This study offers three original key contributions to the literature on algorithmic governance at the workplace. Firstly, the study delivers a thorough legal-sociological theoretical analysis of workplace AI use, suggesting that legal analysis should be enforced by institutional, organizational, and workers’ rights understanding. Secondly, the study advances methodologically by developing an interdisciplinary legal-organizational analysis (LOA) complemented by broadening existing scholarship with a comparative legal analysis of different regulatory systems at a global level, while discussing their practical implications for the enactment of workers’ rights in the context of deploying AI technologies. Thirdly, the study enriches the literature, generating policy-relevant insights that add to regulatory perspectives on the need to align workforce AI governance, technological innovation, and market competitiveness with workers’ rights and data protection.

## Data Availability

The original contributions presented in the study are included in the article/supplementary material, further inquiries can be directed to the corresponding author.
